# Increased risk of COVID-19 mortality rate in *IFITM3* rs6598045 G allele carriers infected by SARS-CoV-2 delta variant

**DOI:** 10.1186/s40246-022-00434-8

**Published:** 2022-11-19

**Authors:** Melika Gholami, Fatemeh Sakhaee, Fattah Sotoodehnejadnematalahi, Mohammad Saber Zamani, Iraj Ahmadi, Enayat Anvari, Abolfazl Fateh

**Affiliations:** 1 Department of Biology, Science and Research Branch, Islamic Azad University, Tehran, Iran; 2 Department of Mycobacteriology and Pulmonary Research, Pasteur Institute of Iran, Tehran, Iran; 3 Immunoregulation Research Center, Shahed University, Tehran, Iran; 4 Department of Physiology, School of Medicine, Ilam University of Medical Science, Ilam, Iran; 5 Microbiology Research Center (MRC), Pasteur Institute of Iran, Tehran, Iran

**Keywords:** Interferon-induced transmembrane protein 3, Coronavirus disease 2019, Severe acute respiratory syndrome coronavirus 2 variants, qPCR Ct values

## Abstract

**Background:**

The interferon-induced transmembrane-protein 3 (IFITM3) is a vital component of the immune system's defense against viral infection. Variants in the *IFITM3* gene have been linked to changes in expression and the risk of severe Coronavirus disease 2019 (COVID-19). This study aimed to investigate whether *IFITM3* rs6598045, quantitative polymerase chain reaction (qPCR) cycle threshold (Ct) values, and severe acute respiratory syndrome coronavirus 2 (SARS-CoV-2) variants are associated with an increased mortality rate of COVID-19.

**Methods:**

The genotyping of *IFITM3* rs6598045 polymorphism was analyzed using the amplification refractory mutation system-polymerase chain reaction in 1342 recovered and 1149 deceased patients positive for SARS-CoV-2.

**Results:**

In this study, *IFITM3* rs6598045 G allele as minor allele frequency was significantly more common in the deceased patients than in the recovered ones. Furthermore, the highest mortality rates were observed in Delta variant and lowest qPCR Ct values. COVID-19 mortality was associated with *IFITM3* rs6598045 GG and AG in Delta variant and *IFITM3* rs6598045 AG in Alpha variant. A statistically significant difference was observed in the qPCR Ct values between individuals with GG and AG genotypes and those with an AA genotype.

**Conclusion:**

A possible correlation was observed between the mortality rate of COVID-19, the G allele of *IFITM3* rs6598045, and SARS-CoV-2 variants. However, large-scale research is still required to validate our results.

**Supplementary Information:**

The online version contains supplementary material available at 10.1186/s40246-022-00434-8.

## Introduction

One of the classical models showing the cause of a disease can be the epidemiological triad of host, environment, and agent. To put it another way, the interaction between the host, the agent, and the mode of exposure (environment) determines the severity of a sickness (host susceptibility and host response to the infectious agent). The recent pandemic of severe acute respiratory syndrome coronavirus 2 (SARS-CoV-2) has shown that significant variability in both virus and host can result in a wide range of clinical outcomes. Early studies on the severity of symptoms tended to concentrate on host characteristics including gender, age, and blood type [1, 2].

As the pandemic spread, more information was obtained about host genetic susceptibility, the impact of the geographical location and virus mutations in severe clinical outcomes [3]. Efforts are being made to forecast the patient's outcomes using computational models built with demographic, genetic, and phenotypic data to tailor patient therapy and manage resources [4].

Early detection of people who are more likely to develop severe symptoms can help maintain life and health. It has been demonstrated that increased mortality is correlated with estimates of viral load at admission, SARS-CoV-2 variants, and host genetic factors including some single nucleotide polymorphisms (SNPs) in Toll-like receptor 3 (*TLR3*), angiotensin-converting enzyme 2 (*ACE2*), transmembrane serine protease 2 (*TMPRSS2*), interferon lambda 3/4, tripartite motif-containing 22, and interferon regulatory factor 7 (*IRF7*) [3, 5–9].

It has been demonstrated that interferon-induced transmembrane proteins (IFITMs), a group of viral restriction proteins, prevent the spread of enveloped RNA viruses including Ebola virus, dengue virus, coronaviruses and influenza. IFITM1, 2 and 3, which are activated in response to type I and II interferon stimulation, are the initial line of defense against pathogen infection [10]. IFITM2 and IFITM3 prevent viruses from entering the cells through late endocytic compartments because they are localized to early and late endocytic vesicles and lysosomes, respectively [11].

Many genotypes have been linked to the increased severity of several viruses. In particular, the most investigated allele is the C variant of *IFITM3* rs12252. The *IFITM3* rs12252 C allele was found in a statistically significant number of hospitalized participants with H1N1 influenza and Coronavirus disease 2019 (COVID-19) [12, 13].

The *IFITM3* rs6598045 allele, situated on the proximal promoter of the *IFITM3* gene, is correlated with transcriptional efficiency via the binding ability of the transcription factor TFII-I and is a novel potential SNP associated with susceptibility to the 2009 pandemic H1N1 influenza A infection [14]. Despite the fact that the 2009 pandemic H1N1 influenza A virus and SARS-CoV-2 have different viral receptors and binding proteins, both viruses share the same target cell, similar respiratory symptoms, and co-morbidities associated with severe infection [15].

Based on the important risk factors for COVID-19 severity, we evaluated the effects of *IFITM3* rs6598045, SARS-CoV-2 variants, and quantitative polymerase chain reaction (qPCR) cycle threshold (Ct) values on COVID-19 mortality.

## Materials and methods

### Sample selection of patient

This retrospective study was performed on patients selected from Ilam University of Medical Sciences in three peaks of COVID-19 infection (Alpha, Delta, and Omicron BA.5) from October 2020 to January 2022. Real-time reverse transcription polymerase chain reaction (rtReal time-PCR) was used to detect SARS-CoV-2 genome in pharyngeal swab specimens. All rtReal time-PCR tests were conducted by expert personnel under the same conditions regarding sampling time, the RNA extraction kit, and the real-time PCR kit.

Only 2,491 participants out of a total of 12,316 patients were considered eligible to participate in the study according to inclusion criteria: (1) providing consent before taking part in the study; (2) having same ethnic background and Iranian nationality; (3) having positive rtReal time-PCR test results and being selected from only one hospital; (4) The patients had no previous history of SARS-CoV-2 infection; (5) having no underlying comorbidities such as chronic obstructive pulmonary disease, cystic fibrosis, cancer, pregnancy, human immunodeficiency virus (HIV), obesity, heart disease, kidney disease, liver disease, and diabetes.

The studied patients were categorized into two groups. Group 1 included recovered patients with any of the signs and symptoms of COVID-19 such as headache, fever, sore throat, muscle pain, malaise, loss of taste and smell, nausea, and diarrhea, but without dyspnea, respiratory distress, or abnormal chest imaging and those with oxygen saturation (SpO_2_) levels below 94% and displayed signs of lower respiratory illness during clinical evaluation or imaging. These patients were outpatients and were not admitted to the hospital, and samples were taken from them when visiting the hospital.

Group 2 included deceased patients with the following features: SpO_2_ below 94%, a PaO_2_/FiO_2_ ratio below 300 mm Hg, a respiratory rate above 30 breaths per minute, lung infiltrates above 50%, septic shock, multiple organ dysfunction, and respiratory failure necessitating the use of mechanical ventilation.

All clinical parameters including aspartate aminotransferase (AST), alanine aminotransferase (ALT), alkaline phosphatase (ALP), low-density lipoprotein (LDL), high density lipoprotein (HDL), cholesterol, triglyceride (TG), uric acid, serum creatinine, fasting blood glucose (FBS), white blood cells (WBC), platelets, C-reactive protein (CRP), erythrocyte sedimentation rate (ESR), thyroid-stimulating hormone (TSH), thyroxine (T4), triiodothyronine (T3), real-time PCR cycle threshold (*C*_*t*_) values, and, 25-hydroxyvitamin D were obtained from the electronic medical record system during pre-hospitalization visits.

### Genomic DNA extraction and *IFITM3* rs6598045 genotyping

Approximately 10 ml of blood samples were collected from infected patients in EDTA tubes. Ficoll density gradient centrifugation (Ficoll-Paque PLUS, GE Healthcare, USA) was used to isolate peripheral blood mononuclear cells (PBMCs) and then the PBMCs were kept at −20 °C until the experiment.

The genomic DNA was extracted from the blood samples with a commercial kit (High Pure PCR Template Preparation Kit, Roche Diagnostics Deutschland and GmbH, Mannheim, Germany), according to the manufacturer's instructions. The yield of DNAs was determined using NanoDrop spectrophotometers (Thermo Scientific, USA), and the quality of DNAs was validated using electrophoresis gel.

The in-house amplification refractory mutation system–polymerase chain reaction (ARMS–PCR) assay was used for *IFITM3* rs6598045 genotyping. The *IFITM3* rs6598045 5′ ARMS primers were constructed with one base mismatch at the 3′ end. The “A” and “G” allele reverse primers were 5´-TCTTAGCCCTCAGCCCCACT-3´ and 5´-TCTTAGCCCTCAGCCCCACC-3´, respectively, and the forward primer was 5´-GCACCCTCTGAGCATTCCCTG-3´. The PCR product size was 490 bp. Moreover, as an internal control, we applied an 827 bp product with the primer sets of 5´-GCACCCTCTGAGCATTCCCTG-3´ and 5´-CACAGTGACGGTTATGGGAGACG-3´ (Additional file 1: Figure S1). The PCR test was carried out by initial denaturation at 95 °C for 18 min, followed by 40 cycles of 95 °C for 20 s, 63 °C for 25 s, 72 °C for 25 s, and final extension at 72 °C for 10 min.

The 890 bp PCR products were sequenced using the Sanger method on an ABI 3500 DX Genetic Analyzer (ABI, Thermo Fisher Scientific, and Waltham, MA, USA) to verify the ARMS-PCR results. MEGA Version 11.0 (https://www.megasoftware.net/) was used to analyze the raw sequencing data (Additional file 1: Figure S2).

### Statistical analyses

The categorical variables as number (%) and continuous variables (numerical variables) as mean ± standard division (SD) were summarized using SPSS version 22.0 (SPSS, Inc, Chicago, IL, USA). The Shapiro–Wilk test was used to evaluate the normality assumptions for all numerical variables. The Mann–Whitney *U* test was used to compare continuous variables with normally distributed distributions. The Chi-square test was used to compare categorical variables. Independent factors of the likelihood and mortality of COVID-19 were identified using logistic regression-based multivariate models. Also, odds ratio (OR) and associated 95% confidence interval (CI) were calculated**.** An area under the receiver operating characteristic curve (AUC-ROC) analysis was used to assess the impact of *IFITM3* rs6598045 on COVID-19 mortality. A *P*-value of < 0.05 was regarded statistically significant, and all tests were two-tailed. The online SNPStats tool was used to calculate the allelic frequency of the chosen variant to determine the minor allele frequency (MAF), four inheritance models (dominant, codominant, overdominant, and recessive), and the Hardy–Weinberg equilibrium (HWE). The optimal model was determined using the Akaike Information Criterion (AIC) and the Bayesian Information Criterion (BIC) (http://bioinfo.iconcologia.net/SNPStats).

## Results

### Characteristics of patients

Table 1 lists the characteristics of COVID-19 patients who participated in this study. The current study enrolled 2491 COVID-19 patients, of whom 1149 were in the deceased group and 1342 were in the recovered group. The age distribution was significantly different between recovered (51.0 ± 13.0) and deceased (59.3 ± 10.9) patients (*P* < 0.001). Of the patients, 1308 (52.5%) were male and 1183 (47.5%) were female.

**Table 1:** Comparison of laboratory parameters between deceased and recovered patients infected with COVID-19

Variables	Deceased patients (*n* = 1149)	Recovered patients (*n* = 1342)	*P*-value
Mean age ± SD	59.3 ± 10.9	51.0 ± 13.0	< 0.001*
Gender (male/female)	611/538 (53.2/46.8%)	697/645 (51.9/48.1%)	0.537
ALT, IU/L (mean ± SD) (Reference range: 5–40)	44.5 ± 23.9	33.2 ± 24.3	< 0.001*
AST, IU/L (mean ± SD) (Reference range: 5–40)	36.9 ± 13.0	31.3 ± 15.7	< 0.001*
ALP, IU/L (mean ± SD) (Reference range: up to 306)	201.9 ± 66.3	171.0 ± 90.6	< 0.001*
Cholesterol, mg/dL (mean ± SD) (Reference range: 50–200)	116.6 ± 38.9	121.8 ± 36.3	< 0.001*
TG, mg/dL (mean ± SD) (Reference range: 60–165)	116.6 ± 41.7	131.5 ± 61.5	< 0.001*
LDL, mg/dL (mean ± SD) (Reference range: up to 150)	70.2 ± 35.6	107.1 ± 49.3	< 0.001*
HDL, mg/dL (mean ± SD) (Reference range: > 40)	30.6 ± 10.9	34.4 ± 11.6	< 0.001*
WBC, 10^9^/L (mean ± SD) (Reference range: 4000–10,000)	7544.4 ± 2663.4	7717.4 ± 2897.6	0.347
CRP, mg/L (mean ± SD) (Reference range: < 10 mg/L Negative)	67.3 ± 21.4	56.9 ± 20.9	< 0.001*
ESR, mm/1st h (mean ± SD) (Reference range: 0–15)	54.5 ± 15.6	46.5 ± 15.3	< 0.001*
FBS, mg/dL (mean ± SD) (Reference range: 70–100)	110.7 ± 42.9	105.6 ± 41.3	< 0.001*
Platelets × 1000/cumm (mean ± SD) (Reference range: 140,000–400,000)	186 ± 76	185 ± 65	0.206
Uric acid, mg/dL (mean ± SD) (Reference range: 3.6–6.8)	3.9 ± 1.2	5.6 ± 1.4	< 0.001*
Creatinine, mg/dL (mean ± SD) (Reference range: 0.6–1.4)	1.2 ± 0.3	0.7 ± 0.2	< 0.001*
T3, ng/dL (mean ± SD) (Reference range: 2.3–4.2)	3.1 ± 1.2	2.5 ± 0.8	0.061
T4, mcg/dL (mean ± SD) (Reference range: 5.6–13.7)	8.9 ± 4.6	8.3 ± 3.3	0.091
TSH, mu/L (mean ± SD) (Reference range: 0.4–4.5)	3.5 ± 1.4	3.3 ± 1.2	0.384
25-hydroxy vitamin D, ng/mL (mean ± SD) (Normal range: 21–150)	25.3 ± 10.1	35.9 ± 13.4	< 0.001*
SARS-CoV-2 variants			< 0.001*
Alpha	396 (48.1%)	427 (51.9%)	
Delta	664 (75.3%)	218 (24.7%)	
Omicron BA.5	89 (11.2%)	697 (88.8%)	
*IFITM3* rs6598045			< 0.001*
AA	190 (17.5%)	897 (82.5%)	
AG	508 (60.6%)	330 (39.4%)	
GG	451 (79.7%)	115 (20.3%)	

*COVID-19* coronavirus disease 2019, *ALT* alanine aminotransferase, *AST* aspartate aminotransferase, *ALP* alkaline phosphatase, *TG* triglyceride, *LDL* low density lipoprotein, *HDL* high density lipoprotein, *WBC* white blood cells, *CRP* C-reactive protein, *ESR* erythrocyte sedimentation rate, *FBS* fasting blood glucose, *T3* triiodothyronine, *T4* thyroxine, *TSH* thyroid-stimulating hormone, *SD* standard deviation, *SARS-CoV-2* severe acute respiratory syndrome coronavirus 2, *IFITM3* interferon-induced transmembrane protein 3.

*Statistically significant (< 0.05)

Increased disease mortality was associated with elevated levels of Cr (*P* < 0.001), CRP (*P* < 0.001), ALT (*P* < 0.001), ALP (*P* < 0.001), AST (*P* < 0.001), ESR (*P* < 0.001), and FBS (*P* < 0.001), low levels of cholesterol (*P* < 0.001), TG (*P* < 0.001), LDL (*P* < 0.001), HDL (*P* < 0.001), 25-hydroxyvitamin D (*P* < 0.001) and uric acid (*P* < 0.001).

### Comparing qPCR ***C***_***t***_ values of recovered and deceased patients

**Fig. 1:**
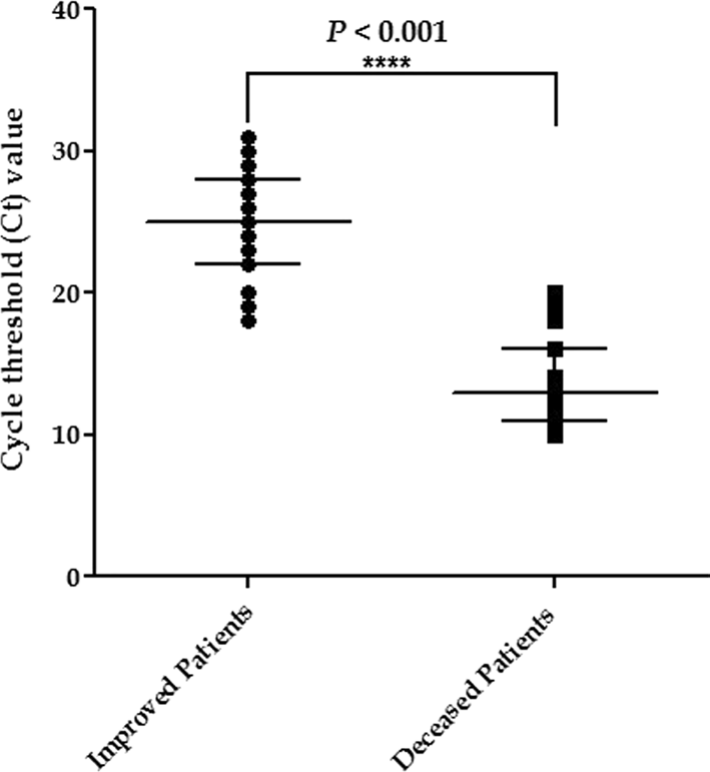
The PCR Ct values in deceased and improved patients

As indicated in Fig. 1, the deceased patients group had a significantly lower observed Ct value (14.2 ± 3.7) than the recovered patients group (24.7 ± 3.8), with a difference of 10 cycles (24.7 vs. 14.2 cycles). The qPCR Ct values were found to be different in SARS-CoV-2 variants. The mean qPCR Ct values of Delta, Alpha, and Omicron BA.5 were 16.6 ± 5.8, 19.9 ± 6.4, and 23.4 ± 5.1, respectively.

### Relationship between *IFITM3* rs6598045 and COVID-19 Mortality

Patients with the *IFITM3* rs6598045 GG and AG genotypes showed a considerably greater COVID-19 death rate compared to patients with other genotypes. Also, patients recovering from COVID-19 had the *IFITM3* rs6598045 AA genotype (Table 1).

0.21The inheritance model analysis results of *IFITM3* rs6598045 among the studied groups are indicated in Table 2. Comparison between the deceased and recovered patients revealed that the codominant inheritance model (with the lowest AIC and BIC values) was the best fit-model for *IFITM3* rs6598045. In this model, the *IFITM3* rs6598045 GG genotype was correlated with an increased risk of COVID-19 mortality (*P* < 0.0001, OR 18.51, 95% CI 14.31–23.96). Also, after adjusting for gender and frequency of the *IFITM3* rs6598045 polymorphism, COVID-19 mortality was associated with the *IFITM3* rs6598045 GG (OR 19.02, 95% CI 13.24–27.32 in male vs. OR 19.06, 95% CI 13.09–27.75 in female) and AG (OR 7.69, 95% CI 5.71–10.36 in male vs. OR 7.24, 95% CI 5.34–9.81 in female).

**Table 2:** *IFITM3* rs6598045 association with COVID-19 mortality

Model	Genotype	Groups		OR (95% CI)	*P*-value	AIC	BIC
		Recovered patients (%)	Deceased patients (%)				
Allele	A	2124 (79.0)	888 (39.0)	–	–	–	–
	G	560 (21.0)	1410 (61.0)	–	–	–	–
Codominant	A/A	897 (66.8)	190 (16.5)	1.00	< 0.0001*	2710.2	2733.5
	A/G	330 (24.6)	508 (44.2)	7.27 (5.90–8.96)			
	G/G	115 (8.6)	451 (39.2)	18.51 (14.31–23.96)			
Dominant	A/A	897 (66.8)	190 (16.5)	1.00	< 0.0001*	2766.9	2784.4
	A/G-G/G	445 (33.2)	959 (83.5)	10.17 (8.39–12.34)			
Recessive	A/A-A/G	1227 (91.4)	698 (60.8)	1.00	< 0.0001*	3098.5	3115.9
	G/G	115 (8.6)	451 (39.2)	6.89 (5.51–8.63)			
Overdominant	A/A-G/G	1012 (75.4)	641 (55.8)	1.00	< 0.0001*	3336.8	3354.3
	A/G	330 (24.6)	508 (44.2)	2.43 (2.05–2.88)			

		Recovered patients	Deceased patients	All patients			
Minor allele frequency (G)		0.21	0.61	0.41	–	–	–

*IFITM3*: Interferon-induced Transmembrane Protein 3; COVID-19: Coronavirus disease 2019; OR: Odds ratios; CI: confidence intervals; AIC: Akaike information criterion; BIC: Bayesian information criterion

*Statistically significant (< 0.05)

In both recovered (*P* < 0.001) and deceased (*P* = 0.021) patients, *IFITM3* rs6598045 genotypes were found to be incompatible with HWE. It is important to note that HWE might not be met in the case sample, implying that SNP is associated with the disease. Moreover, MAF (G-allele) was 0.61, 0.21, and 0.41 in deceased, recovered, and all patients, respectively (Table 2).

**Fig. 2:**
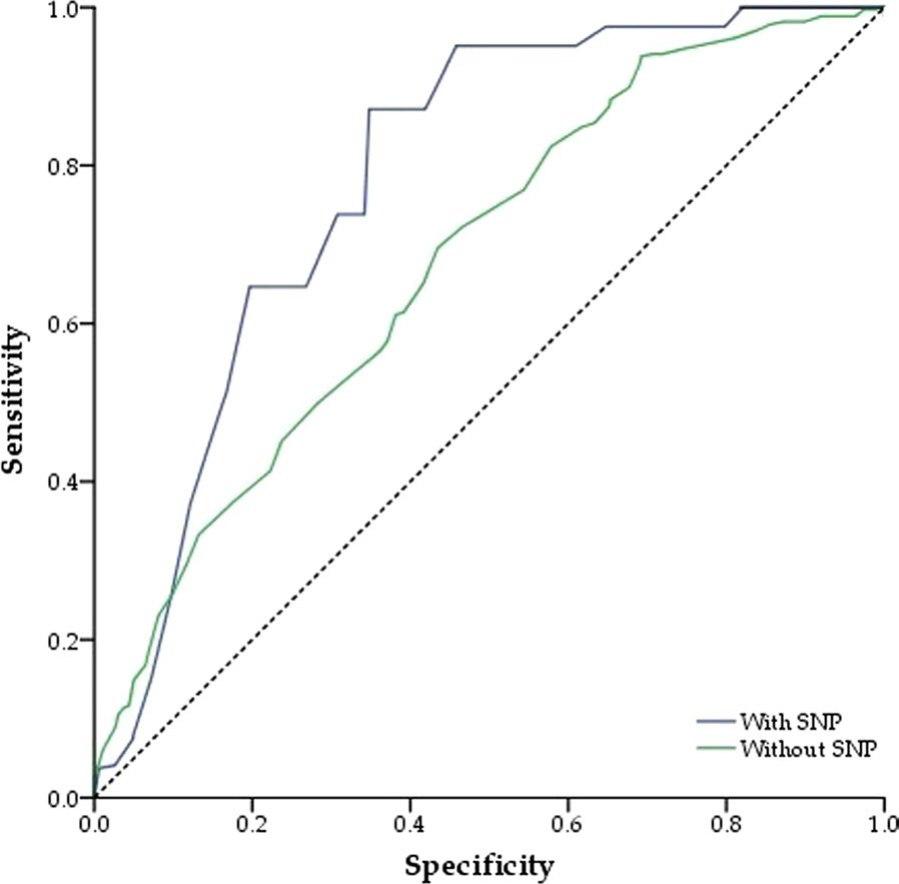
ROC curve for different predictor variable with and without the *IFITM3* rs6598045 genotypes

We utilized logistic regression to generate two distinct predictor variables with and without *IFITM3* rs6598045 genotypes. The AUC-ROC values in the presence and absence of SNPs were 0.845 and 0.567, respectively, indicating that host genetic variables are frequently important for viral infection resolution (Fig. 2).

### Relationship between *IFITM3* rs6598045 genotypes and SARS-CoV-2 Variants

The mortality rate was significantly associated with SARS-CoV-2 variants. The low and high mortality rates were shown in Omicron BA.5 and Delta variants (*P* < 0.001). The frequency of *IFITM3* rs6598045 AA, AG, and GG in Alpha variant was 124 (15.1%), 668 (81.2%), and 31 (3.7%), respectively. In Delta variant, the frequency of *IFITM3* rs6598045 AA, AG, and GG was 259 (29.4%), 112 (12.7%), and 511 (57.9%), respectively. Also, the frequency of *IFITM3* rs6598045 AA, AG, and GG in Omicron BA.5 variant was 704 (89.6%), 58 (7.4%), and 24 (3.0%), respectively.

After adjusting for SARS-CoV-2 variants and *IFITM3* rs6598045 genotypes, COVID-19 mortality was associated with *IFITM3* rs6598045 GG (OR 35.16, 95% CI 8.49–145.54) and AG (OR 2.20, 95% CI 1.59–3.05) in Delta variant and with *IFITM3* rs6598045 AG (OR 4.10, 95% CI 2.58–6.53) in Alpha variant (Table 3).

**Table 3:** *IFITM3* rs6598045 association with SARS-CoV-2 Variants

Variants	Genotype	Recovered patients	Deceased patients	OR (95% CI)
Alpha	A/A	99	25	1.00
	A/G	328	340	4.10 (2.58–6.53)
	G/G	1	12	–
Delta	A/A	101	158	1.00
	A/G	14	98	2.20 (1.59–3.05)
	G/G	115	396	35.16 (8.49–145.54)
Omicron BA.5	A/A	697	7	1.00
	A/G	3	55	–
	G/G	2	22	–

*SARS-CoV-2* severe acute respiratory syndrome coronavirus 2, *IFITM3* interferon-induced transmembrane protein 3, *OR* odds ratios, *CI* confidence intervals

### *IFITM3* rs6598045 polymorphism and qPCR Ct value

**Fig. 3:**
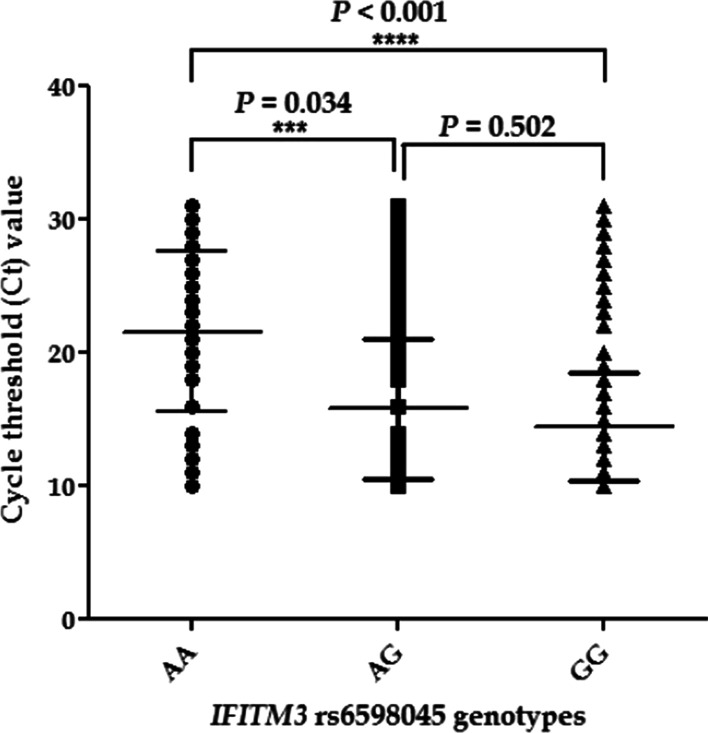
Analysis of PCR Ct values with *IFITM3* rs6598045 genotypes

Next, we investigated the relationship between *IFITM3* rs6598045 polymorphism and viral load. All patients tested positive for the RNA of the SARS-CoV-2 virus. As a semi-quantitative prediction of viral load, the Ct value was utilized. We analyzed the qPCR Ct values, which were conducted when the patients were admitted to the hospital. A lower qPCR Ct value is likely to be related to a higher viral load, while an increased Ct value is likely to be related to a decreased viral load. As depicted in Fig. 3, a statistically significant difference was observed in the qPCR Ct values between individuals with GG (*P* < 0.001) and AG (*P* = 0.034) genotypes and those with an AA genotype, suggesting a probable link between this genotype and an increased viral load. In addition, the Pearson correlation analysis revealed a tendency for the Ct value to decrease in the presence of the G allele (*P* = 0.082).

### Risk factors associated with COVID-19 mortality

The association between risk variables and COVID-19 clinical mortality was studied using a multivariate logistic regression model. The COVID-19 mortality rate was related to mean age, ALT, HDL, LDL, FBS, uric acid, creatinine, ESR, 25-hydroxyvitamin D, real-time PCR Ct values, SARS-CoV-2 variants, and *IFITM3* rs6598045 GG (Table 4).

**Table 4:** Factors associated with deceased patients infected by SARS-CoV-2

Baseline predictors	OR (95% CI)	*P*-value
**Factors**		
Mean age ± SD	0.943 (0.922–0.963)	< 0.001*
ALT, IU/L	0.980 (0.970–0.991)	< 0.001*
HDL, mg/dL	1.044 (1.022–1.066)	< 0.001*
LDL, mg/dL	1.018 (1.012–1.024)	< 0.001*
FBS, mg/dL	0.991 (0.958–0.997)	0.004*
Uric acid, mg/dL	2.015 (1.701–2.387)	< 0.001*
Creatinine, mg/dL	0.069 (0.032–0.152)	< 0.001*
ESR, (mm/1st h)	0.973 (0.957–0.989)	0.001*
25-Hydroxyvitamin D, (ng/Ml)	1.038 (1.016–1.059)	< 0.001*
Real-time PCR Ct values	1.820 (1.685–1.966)	< 0.001*
SARS-CoV-2 variants	2.431 (1.724–3.427)	< 0.001*
*IFITM3* rs6598045 (GG)	0.317 (0.219–0.460)	< 0.001*

*SD* standard deviation, *ALT* alanine aminotransferase, *HDL* high density lipoprotein, *LDL* low density lipoprotein, *FBS* fasting blood glucose, *ESR* erythrocyte sedimentation rate, *CRP* C-reactive protein, *Ct* cycle threshold, *SARS-CoV-2* severe acute respiratory syndrome coronavirus 2, *IFITM3* interferon-induced transmembrane protein 3, *OR* odds ratios, *CI* confidence intervals

*Statistically significant (< 0.05)

## Discussion

We found a statistically significant association between the allele frequency of *IFITM3* rs6598045 and the case mortality rate of COVID-19. To the best of our knowledge, this work is the first to outline a significant association between *IFITM3* rs6598045 SNP and COVID-19 mortality in Iran.

Based on our findings, the *IFITM3* rs6598045 G variation was associated with an increased risk of death and hospitalization due to COVID-19. The MAF (G-allele) for *IFITM3* rs6598045 in this study was 0.41. This amount in the population of other regions was in Asian (0.167), East Asian (0.140), South Asian (0.311), other Asian (0.250), European (0.149), African (0.39), African American (0.303), and Latin American (0.298), as reported in the National Center for Biotechnology Information (NCBI) single nucleotide polymorphism database (https://www.ncbi.nlm.nih.gov/snp/rs6598045).

The frequency of the *IFITM3* rs6598045 G allele was higher in the deceased patients (0.61) than in the recovered ones (0.21). The MAF of rs6598045 indicated a strong association with the COVID-19 mortality rate [16].

By forming a chain-like structure on the cell membrane between IFITM3 protein monomers, it has been hypothesized that the IFITM3 protein physically prevents the endocytosis of a number of viruses [13, 17]. Therefore, the IFITM3 protein's ability to combat viruses is greatly influenced by SNPs that impact the protein's function and amount of expression. The severity of COVID-19 is linked to the *IFITM3* rs12252, which is associated with the truncated form of the IFITM3 protein [13]. The *IFITM3* rs34481144 allele is positioned on the regulation region of the *IFITM3* gene and the CCCTC-binding factor. The severity of the 2009 H1N1 influenza pandemic is correlated with the rs34481144 allele, which modifies the binding affinity of the transcription factor of the *IFITM3* gene [17, 18].

Recent investigations have also revealed that two regulatory SNPs, *IFITM3* rs34481144 and rs6598045, located in the promoter area affect promoter activity by modifying the binding affinity of the transcription factors CTCF and TFII-I, respectively. These two regulatory SNPs impacted the *IFITM3* gene transcriptional activity and were associated with the severity and susceptibility to viral infection such as COVID-19 infection and influenza A virus [14, 17]. The mechanism of action of the three SNPs and their relationship with viral infection are still unknown. Additionally, the difference in genotype and *IFITM3* SNP allele distributions based on ethnic background has not yet been discovered [16]. Kim et al. indicated that three SNPs within the *IFITM3* gene had varying *p*-values. Although these SNPs are positioned within 1 kb of one another, their innate immune response mechanisms are vastly distinct. However, *IFITM3* rs6598045 appears to play a more dominant role in COVID-19 severity than the other two SNPs [16].

In the current study, the highest mortality rate was shown in Delta variant. A meta-analysis showed that regarding hospitalization, ICU admission, and mortality, Alpha, Beta, Gamma, and Delta variants were all more dangerous than the wild-type virus, with Beta and Delta variants having a higher risk than Alpha and Gamma variants. Interestingly, the random effects of Beta variant relative to the wild-type virus for hospitalization, severe disease, and mortality rates are 2.16-, 2.23-, and 1.50-fold, respectively; these effects are 2.08-, 3.35-, and 2.33-fold, respectively, for Delta variant relative to the wild-type virus [6].

The COVID-19 mortality rate in Delta variant was associated with the *IFITM3* rs6598045 G allele in this study. One of the potential reasons for the high mortality in Delta variant is the presence of this allele among the patients. However, more studies are needed to confirm this hypothesis.

We found an independent relationship between the qPCR Ct value and COVID-19 mortality, in which a lower qPCR Ct value was linked to a higher risk of death. The qPCR Ct levels may provide early risk classification in COVID-19 patients. Indeed, the relationship between qPCR Ct and COVID-19 mortality has already been detected in a systematic evaluation of 18 reports, with the majority indicating positive correlations, and has also been documented in a more recent narrative review. A qPCR Ct value of less than 20 is typically termed "very infective” [19–21].

A statistically significant difference was observed in the qPCR Ct values between individuals with *IFITM3* rs6598045 GG and AG genotypes and those with an AA genotype, suggesting a probable link between this genotype and an increased viral load in our study. It seems that the *IFITM3* rs6598045 G allele causes the severity of the COVID-19 infection by increasing the amount of viral load. It is conceivable that patients with *IFITM3* rs6598045 GG reveal a decreased ability in clearance of SARS-CoV-2, correlated with the defective inflammatory pathway upregulation.

In this study, one important factor associated with COVID-19 mortality was low level of 25-hydroxyvitamin D. Studies show that people with 25-hydroxyvitamin D deficiency are more prone to lower respiratory tract infections. They are 117% more likely to need oxygen therapy and 217% more likely to need mechanical ventilation than people with sufficient 25-hydroxyvitamin D [22]. Since 25-hydroxyvitamin D has been shown to play a part in a number of infectious diseases that affect the respiratory system, it was hypothesized that 25-hydroxyvitamin D would also play a part in the SARS-CoV-2 infection. The enhanced production of T helper 1 (Th1) pro-inflammatory cytokines (leading to a cytokine storm) is one of the mechanisms through which SARS-CoV-2 damages lung tissue and causes acute respiratory failure [23]. Vitamin D reduces cytokine storm by shifting pro-inflammatory Th1 and Th17 responses to anti-inflammatory Th2 and regulatory T cell responses [24]. According to the evidence presented above, vitamin D may play an important role in preventing, alleviating, or treating COVID-19 infection signs, such as severe pneumonia [25].

The use of qPCR Ct values for COVID-19 mortality is one of the study's limitations. The diversity in sample collection per patient, where some people referred to clinics early while others referred to clinics later after symptoms became more severe, is another limitation of utilizing the PCR Ct value. The relationship between the COVID-19 mortality rate with *IFITM3* rs6598045 and qPCR Ct values was investigated in three variants but not in other variants due to the non-availability of data.

In conclusion, the *IFITM3* rs6598045 gene polymorphism was associated with COVID-19 mortality. Similarly, the *IFITM3* rs6598045 G allele in both genders was associated with COVID-19 mortality. Furthermore, the highest mortality rate and the lowest qPCR Ct values were shown in Delta variant. COVID-19 mortality was associated with *IFITM3* rs6598045 GG and AG in Delta variant and *IFITM3* rs6598045 AG in Alpha variant. A statistically significant difference was observed in the qPCR Ct values between individuals with GG and AG genotypes and those with an AA genotype. In future, it will be crucial to determine whether the *IFITM3* rs6598045 SNP is reliably associated with COVID-19 mortality.

## Supplementary Information

**Additional file 1: Supplementary Figure 1:** IFITM3 rs6598045 ARMS-PCR genotyping. **Supplementary Figure 2:** The sequencing results of IFITM3 rs6598045 genotypes for confirming the ARMS-PCR method.

## Data Availability

All data generated or analyzed during this study are included in this published article.
