# Supplementary material for: Increased risk of COVID-19 mortality rate in IFITM3 rs6598045 G allele carriers infected by SARS-CoV-2 delta variant
Source: Hum Genomics. 2022 Nov 19;16:60. doi: 10.1186/s40246-022-00434-8 (PMC9675951; doi:10.1186/s40246-022-00434-8)
Supplement: Supplementary file 1 — Additional file 1: Supplementary Figure 1: IFITM3 rs6598045 ARMS-PCR genotyping. Supplementary Figure 2: The sequencing results of IFITM3 rs6598045 genotypes for confirming the ARMS-PCR method. [file 40246_2022_434_MOESM1_ESM.docx]

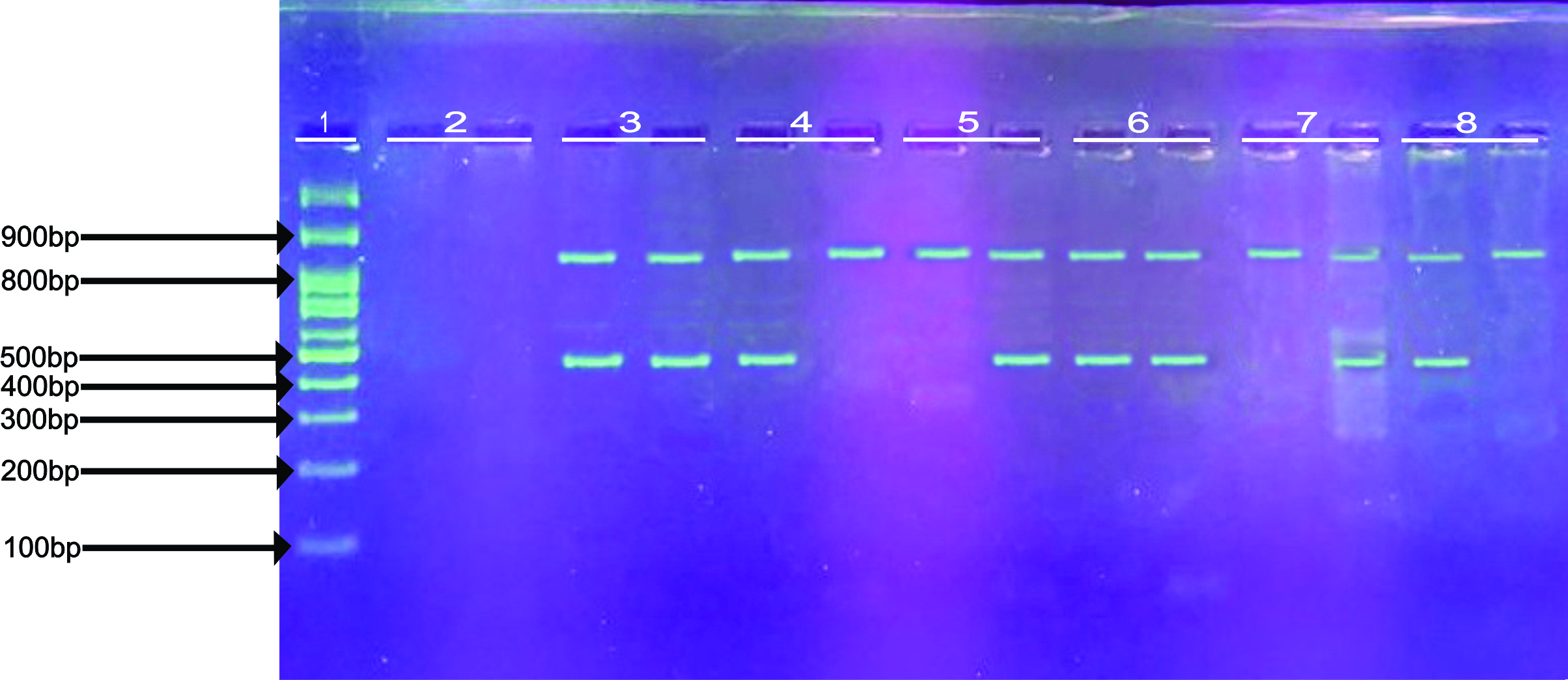


**Supplementary Figure 1:** *IFITM3* rs6598045 ARMS-PCR genotyping. Lane No. 1. DNA marker (100 bp); Lane No. 2 is Negative control, Lane No 3 and 6 are genotype AG (490 bp and 827 bp as an Internal control), Lane No 4 and 8 are genotype AG (490 bp and 827 bp as an Internal control), and Lane No. 5 and 7 are genotype GG (490 bp and 827 bp as an Internal control).


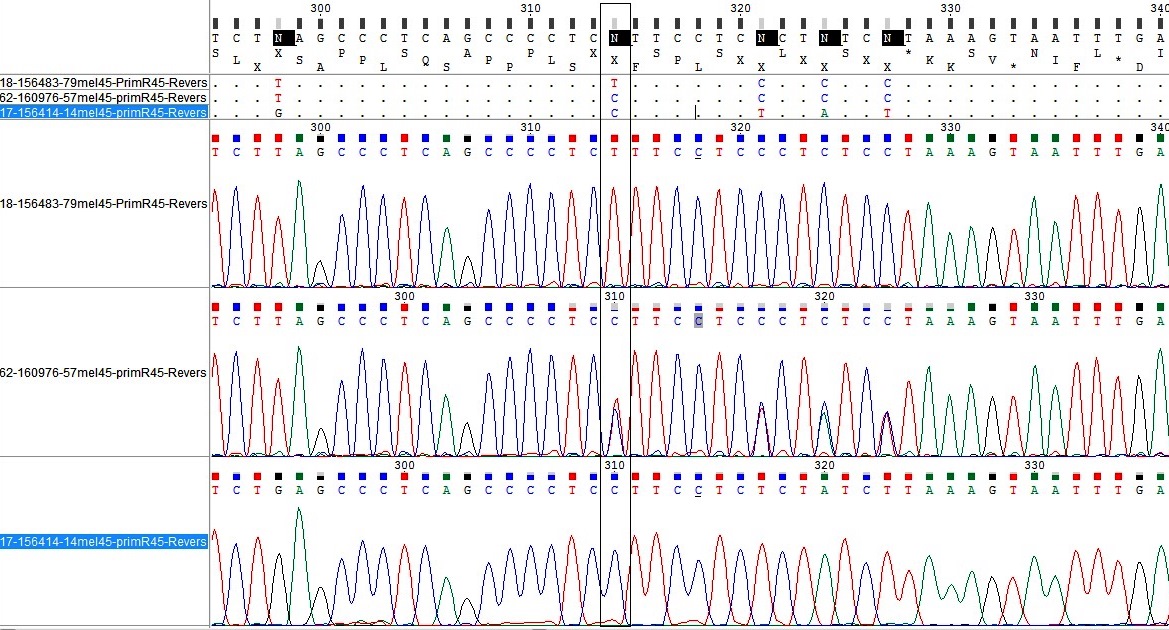


**Supplementary Figure 2:** The sequencing results of *IFITM3* rs6598045 genotypes for confirming the ARMS-PCR method (10% of samples randomly were sequenced)
